# Neuroecology and educational equity: neural regulation, interpersonal synchronization, and social development

**DOI:** 10.3389/fnhum.2026.1810861

**Published:** 2026-04-24

**Authors:** Tal Dotan Ben-Soussan, Leonardo Lamas, Patrizio Paoletti

**Affiliations:** 1 Research Institute of Neuroscience, Education and Didactics (RINED), Patrizio Paoletti Foundation, Assisi, Italy; 2 Ipazia Higher Education Institute, Xgħajra, Malta; 3 Faculty of Physical Education, University of Brasilia, Brasilia, Brazil

**Keywords:** economy, education, EEG, equity, hyperscanning, learning, neuroscience, synchronization

## Introduction

The growing challenges facing neuroscience, education, health, and social equity underscore the need to rethink how human development is conceptualized and supported. Education may function as a key environmental regulator through which biological potential is expressed or compensated for, shaping whether diversity becomes capability rather than inequity (Wilding et al., 2024). In this sense, education can be seen not only as a social and cultural system, but also as a biological regulator, influencing development and gene–environment interactions that shape learning trajectories across the lifespan (Ben-Soussan and Paoletti, 2024). At the biological level, educational and socioeconomic environments can influence gene–environment interactions through epigenetic mechanisms such as DNA methylation, thereby modulating neural plasticity, stress regulation, and human development across development (Meaney and Szyf, 2005; Szyf et al., 2008). Childhood socioeconomic status (SES) shapes neural systems supporting language and executive functions through prenatal influences, parent–child interactions, and cognitive stimulation at home, highlighting how environmental factors drive individual differences in brain development. Related evidences inform policies aimed at reducing SES-related disparities in mental health and academic achievement (Hackman et al., 2010).

**Figure 1:**
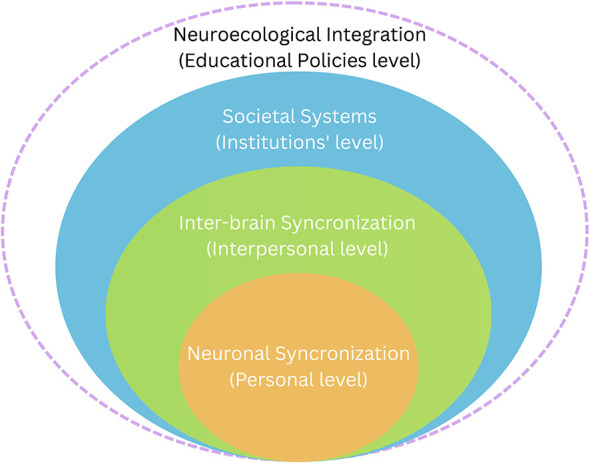
The neuroecological framework starting from brain synchronization to educational policy addressing personal, inter-personal and societal levels.

The International Science and Evidence Based Education Assessment recently examined whether contemporary education systems enable individuals to develop their full potential, consistent with the UN Declaration of Human Rights, and contribute to Sustainable Development Goal 4. Drawing on the work of nearly 300 scientists, it calls for a fundamental shift in educational priorities, from a narrow focus on economic growth toward fostering holistic human flourishing (Singh et al., 2025). In our view, human development requires a multidimensional framework that integrates neurobiological, educational, and socioeconomic systems (Stodden et al., 2023), recognizing that persistent inequalities arise primarily from systemic structures rather than individual effort (La Porta and Zapperi, 2023; Lemke and Sabelli, 2008; Ostrom, 2009). Accordingly, the concept of *Neuroecology*, rooted in the Ecology of Mind (Bateson, 1972), conceptualizes human functioning as an interdependent process in which endogenous brain rhythms, interbrain coupling, and environmental constraints mutually influence one another through ongoing socioeconomic interactions and ecological context. Rather than focusing on isolated cognitive functions of a single individual, this perspective emphasizes how neural, psychobiological, social, and environmental components may interact to shape educational outcomes (Choudhary et al., 2024). This neuro-ecological perspective also provides the conceptual basis for the economical alignment with individual, interpersonal, and social systems (Figure 1).

Educational systems emphasizing *individuality* promote autonomy, creativity, and differentiated talent development, but risk amplifying socioeconomic inequalities and fragmented learning trajectories when such capacities are insufficiently nurtured early in life (Heckman, 2011; Heckman and Mosso, 2014; Moffitt et al., 2011). In contrast, *collective*-oriented systems prioritize shared norms, and coordinated learning environments, fostering equity, which may constrain individual expression if applied rigidly (Bronfenbrenner, 2005; Hofstede, 2001). Thus, an optimal educational model integrates both approaches (Diamond, 2013; Heckman et al., 2006, 2018), prioritizing collective collaboration, developing hot and cold executive functions and self-expression (Heckman et al., 2018; Ostrom, 2009). We shall now detail, what are the related mechanisms, across three levels: personal and interpersonal synchronization in educational contexts, and their alignment shaping educational equity and development.

### Neural synchronization, cognition, and learning

Slow wave neural synchronization, and especially alpha and theta (8–12 and 4–7 Hz, respectively), provide a foundation for cognitive and emotional regulation (Buzsáki, 2006). These slow rhythms coordinate activity across distributed neuronal populations, facilitating perception, memory, and executive function (Paoletti and Ben-Soussan, 2020; Neubauer and Fink, 2009; Başar, 2012; Fries, 2015). Alpha activity in particular is thought to regulate cognition by acting as a top-down thalamocortical gain-control system that filters sensory and internal information. Thus, alpha activity is not only related to different cognition aspects—for example, attention, cognitive flexibility (Ben-Soussan et al., 2013; Klimesch, 2012), but is fundamental also to the perception of the self (D'angelo et al., 2026; Paoletti and Ben-Soussan, 2020). Through their role in shaping bodily awareness, they also contribute to how individuals relate to others and to the social world. When this regulatory system is disrupted by stress, cognitive overload or environmental instability, attention fragments, executive control weakens. As a result, maladaptive biases may dominate, and learning capacity deteriorates. Conversely, embodied practices can restore these rhythms by activating alpha synchronization increasing awareness to bodily sensations, reducing mind-wandering and rumination, thereby strengthening attention, regulation and learning efficiency (Kerr et al., 2013; Lasaponara et al., 2017; Wang et al., 2025). These regulatory mechanisms may provide the neurobiological conditions for interpersonal synchronization.

### Interpersonal synchrony in educational contexts

In recent years, neuroscientific studies have finally started approaching inter-brain synchronization utilizing hyperscanning, examining how neural coupling between students may enable enhanced cooperation and shared attention. For example, (Dikker et al. 2017) examining a group of high school seniors for a semester and recording their brain activity during their regular biology classes, found that students' brainwaves are more in sync with each other when they were more engaged during class. Brain-to-brain synchrony was further reflective of teacher likability, which is important for a positive learning environment (Nam et al., 2020). Another study has further shown that classrooms with high interpersonal synchrony—involving shared emotional attunement, synchronized attention, and cooperative activities—lead to improved learning outcomes and group performance, including math and languages (Chen et al., 2023). More specifically, students with higher scores in Math were found to have stronger inter-brain couplings to all other classmates, whereas the students with higher scores in Chinese were found to have stronger inter-brain couplings to the top students in the class. Importantly, inter-brain synchronization during a cooperative task differed from that observed during competition (Liu et al., 2021). Cooperation increased widespread delta/theta inter-brain amplitude coupling, while competition reduced occipital alpha/beta coupling (Liu et al., 2021). The ability to entrain with others depends on individual rhythmic stability, suggesting that individuals with better self-regulation are more likely to synchronize with peers, forming the foundation for effective collaboration and social intelligence.

In addition to hyperscanning as a methodological approach to studying interpersonal neural dynamics, alpha–mu rhythms index the functional integrity of the mirror neuron system (Fox et al., 2015). As such, their modulation reflects mechanisms that support the healthy development of body awareness and, consequently, empathy and social attunement through efficient self–other mapping and action understanding (Oberman et al., 2008). Atypical modulation or reduced suppression of alpha–mu rhythms has been associated with neurodevelopmental conditions, such as autism spectrum disorder, reflecting alterations in mirror neuron functioning, interpersonal synchrony and social cognition (Oberman et al., 2005, 2008; Dikker et al., 2017). Because interpersonal synchrony depends on individual regulatory stability, educational environments become key contexts where neural, relational, and developmental processes interact.

In the context of educational systems, promoting environments where these neural processes are supported can foster effective social development and empathy among learners (Iacoboni et al., 1999; Iacoboni, 2009). In addition, a growing body of research on preschool interventions targeting executive functions demonstrates that high-quality early childhood learning environments (typically children aged 3–5 years) can enhance working memory, inhibition, and cognitive flexibility, essential skills for cognitive development, social participation, and later academic success (for a recent review see Pastena et al., 2025). Importantly, evidence from low-socioeconomic contexts shows that the most effective interventions are not narrowly cognitive, but relational: movement-based activities, emotional attunement, communication, and well-structured environments play a central role in fostering executive functions and reducing behavioral difficulties (Mavilidi et al., 2025; Stodden et al., 2023).

### Societal development and educational equity

Socioeconomic adversity impairs neural efficiency and cognitive development, often leading to cumulative disadvantage (Amso and Lynn, 2017; Kim et al., 2017). Chronic stress and instability increase physiological noise, making it harder for children to regulate emotions and focus on learning tasks. In addition, children from lower socioeconomic backgrounds exhibit altered neural activation patterns (D'angiulli et al., 2012), which can affect both cognitive resources and neural efficiency (Kamgang et al., 2023). While structured, emotionally supportive, and physically engaging interventions can enhance executive functions across socioeconomic groups and in the general population—highlighting the importance of a multidimensional translational approach (Pastena et al., 2025)—educational inequity reflects not only social disadvantage, but may also mirror disruptions in the developmental regulation of attention, emotion, and learning. These disruptions therefore need to be addressed in a coordinated manner at both the individual and societal levels.

Dynamic models of educational choice show that education yields substantial benefits, including improved mental health, reduced welfare dependency, and increased civic engagement, with effects that are often strongest for individuals from disadvantaged backgrounds (Heckman et al., 2018). These findings align closely with neuroscience evidence demonstrating that learning depends on the development of attentional control and self-regulation, processes that emerge early, are highly plastic, and are profoundly shaped by environmental conditions. In this context, education operates as a dynamic intervention: early investments alter developmental trajectories by opening future learning options and strengthening regulatory capacities, whereas late, uniform expansions (e.g., indiscriminate college promotion) often yield low marginal returns for those least prepared to benefit. From this perspective, educational inequality is not merely a distributional problem, but a neurodevelopmental one, rooted in unequal opportunities to cultivate the attentional, emotional, and bodily foundations of learning. Effective educational policy should therefore move beyond test scores and isolated cognitive outcomes toward early interpersonal interventions that align economic efficiency with the science of learning and human development (Heckman, 2011).

## Discussion: from neuroscience and education to Spherical Economy

The Neuroecology perspective provides a theoretical framework for understanding learning and development as outcomes of interconnected systems, such as electrophysiological, psychobiological, interpersonal, socioeconomical and educational levels. By aligning aims at the individual, group, and societal levels, education can serve as an intervention to promote equity and sustainable development. Within this framework, the *Spherical Economy* (Ben-Soussan and Paoletti, 2026) emerges as the natural evolution of the Spherical Model of Consciousness—a philosophical, scientific and humanistic vision that unites inner life, society and sustainable development (Paoletti, 2008; Paoletti et al., 2024). In an age marked by crisis and fragmentation, it calls for a paradigm shift: from the logic of competition to the logic of cooperation. It advances beyond both linear and circular economic models (Michelini et al., 2017): while the latter emphasizes the sustainable management of materials (reduce, reuse, and recycle), the Spherical Economy introduces a third, “spherical” dimension that incorporates the human being—along with their consciousness, ethical orientation, and capacity for intentional action—at the center of economic processes. In this sense, it promotes a process of re-humanization, shifting from profit-maximization toward the restoration of meaning and relational value within economic activity. Grounded in systems theory, contemplative science, and neuroscience, this approach aligns with neurobiological evidence on social cognition, emotional regulation, and prosocial behavior, supporting a paradigm shift from competitive, scarcity-based logics (*mors tua, vita mea*) to cooperative and interdependent ones (*vita tua, vita mea*). The model emphasizes shared responsibility and awareness as drivers of preventive, rather than merely reparative, economic action, encouraging individuals and organizations to adopt choices that generate positive impacts for both self and others. At the organizational level, it reconfigures the firm from a hierarchical structure into a networked system of relationships, fostering collaboration among stakeholders and recognizing systemic interdependence. Within this framework, value is redefined not only in economic terms but also in social and human terms, with collective wellbeing and even happiness emerging as legitimate dimensions of business models. Ultimately, the Spherical Economy represents an ethical and adaptive evolution of existing paradigms, aiming to create sustainable development through the continuous integration of individual, group, and collective dimensions, as well as inner states and external actions. In this sense, the Spherical Economy is not only a conceptual or ethical framework, but may also be grounded in neurobiological mechanisms. Specifically, individual neural regulation (e.g., alpha/theta synchronization) may support interpersonal synchrony and prosocial behavior, which in turn may contribute to cooperative and equitable socio-economic systems (Czeszumski et al., 2020; Valencia and Froese, 2020). While the extension of neural and interpersonal coordination mechanisms from individual interactions to group-level and, ultimately, institutional dynamics through repeated social engagement is indirect, converging evidence suggests that patterns of interpersonal synchrony may, over time, contribute to the emergence of shared norms, cooperation, and coordinated behavior at larger social scales.

Accordingly, understanding human intelligence, performance and success requires studying how neural systems interact with social networks, shared knowledge systems, and communal practices, shifting neuroscience toward models that integrate individual brain processes with distributed, group-level cognition (Sloman et al., 2021). Aligning neural rhythms between individuals has been associated with collaborative learning and social intelligence, supporting the argument that educational systems must foster interpersonal synchrony to optimize shared performance. This perspective supports a necessary shift in educational policy, from approaches that focus narrowly on individual cognitive performance to systemic frameworks informed by interpersonal studies (e.g., hyperscanning), that explicitly address how socioeconomic inequalities shape bodily regulation, attention, learning opportunities, and collaboration. As these interpersonal neural mechanisms do not develop in a social vacuum, neuroeducational interventions must foster them at every level, from individuals to institutions, to promote adaptive learning systems that support long-term social mobility and equity. In this sense, the current opinion paper does not aim to provide a systematic review; instead, it advances an integrative conceptual framework that connects neuroscientific evidence with educational practice and socioeconomic policy.

To empirically examine the proposed neuroecological framework, multi-level longitudinal designs may be implemented across various educational contexts, with the school setting representing a particularly suitable environment. At the personal level, longitudinal changes in alpha/theta synchronization and executive functions could be assessed through repeated-measures pre- and post-intervention designs using EEG and standardized behavioral tasks. At the interpersonal level, hyperscanning during classroom activities can quantify inter-brain synchronization and classroom coordination correlates (Chen et al., 2023), related among other things to cooperative and competative activities (Nam et al., 2020). At the societal level, conducting large–scale stratified cluster randomized controlled trials, comparing an embodied learning-based curriculum vs. a standard curriculum, enables a socially relevant test of moderated mediation. Specifically, this will allow determining if neural synchronization mediates improvements in executive function, social cohesion, and academic outcomes, and whether this mechanism is particularly impactful within lower-SES contexts. The empirical results may, in turn, support multilevel computational simulations of these complex dynamics (Kasman et al., 2025). Gender and inter-cultural variables may be particularly relevant (Althoff et al., 2017).

Cohort designs can test the hypothesis that embodied learning actively reshapes socioeconomic trajectories rather than merely operating within existing constraints. This shift may occur through enhanced self-awareness (London et al., 2023) and, consequently, increased prosocial behaviors (Liu et al., 2024; Lewis et al., 2021). Interestingly, while self-awareness is typically related to one's psychological changes, the persistent failure to identify a definitive neural signature for the subjective experience (Cogitate Consortium et al., 2025) lead to hypothesize about a collective synergy as a source for broader systemic coherence, according to which the social order is defined beyond purely a product of “brain-to-brain” physical interaction (Wahbeh et al., 2022). The topic has rarely been examined by exploratory studies investigating how self-awareness might emerge as an ordering principle within a broader, non-local environment (Plonka et al., 2026; Carpenter et al., 2021). Although still preliminary, these are exemplar cases of novel empirical frameworks attempting to approach the extent to which shared prosocial orientations and equity-related values emerge and cohere.

The neuroecological framework suggests that educational equity and sustainable development depend on cultivating neural regulation. This process requires educational systems to align individual brain development with interpersonal and social integration, consistent with the principles of the Spherical Economy.
